# Cost savings associated with a nurse driven mobilization protocol for recovery after cranial tumor resection

**DOI:** 10.1007/s00701-025-06641-1

**Published:** 2025-09-02

**Authors:** David Zarrin, Shivani Baisiwala, Jonah Im, Keshav Goel, Myungjun Ko, Sonia Wang, Humza Zubair, Alexander Valenzuela, Tristan Bennett, Dupre Orr, Won Kim

**Affiliations:** 1 UCLA Department of Neurosurgery, David Geffen School of Medicine, UCLA, Los Angeles, CA USA; 2 David Geffen School of Medicine, UCLA, Los Angeles, USA

**Keywords:** Nurse-driven mobilization, Craniotomy, Cost savings

## Abstract

**Background and objectives:**

Neurosurgical procedures can be associated with significant post-operative pain and diminished ability to ambulate or transfer, frequently requiring evaluation by physical / occupational therapy (PT/OT) to ensure appropriate discharge disposition. Owing to high demand for PT/OT services across surgical subspecialities, PT/OT evaluation often bottlenecks disposition. Through our established cranial Enhanced Recovery After Surgery (ERAS) pathway, Neurosurgery Enhanced Recovery Value and Safety (NERVS), our institution employs a nurse-driven mobilization component during post-operative recovery. Here, we report our eight-year experience with a unique institutional NERVS program.

**Methods:**

This is a retrospective observational cohort study. We created a database of elective cranial tumor resections from 2017–2024. Patient demographics, hospitalization metrics, pain levels, and medications were extracted via chart review. Patients discharged home were selected for accurate comparison of outcomes. Analyses were performed in MATLAB.

**Results:**

We identified 1,594 elective craniotomy patients for analysis: 1,059 (66%) entered NERVS, 834 (52%) passed NERVS, 225 (14%) failed NERVS, and 535 (34%) did not enter. Among propensity-matched patients with a post-operative ICU LOS < 1 day, NERVS and no-NERVS groups did not differ in age (53.7 vs 55.1 years, *p* = 0.82), procedure duration (3.9 vs 3.6 h, *p* = 0.08), racial composition (*p* = 0.24–1), or tumor type (*p* = 0.23–0.89). Hospital LOS was significantly shorter among NERVS vs non-NERVS patients (2.9 vs 4.6 days, *p* < 0.001); this was associated with a reduction in total hospital charges on a per-patient basis (-$26,040, *p* < 0.001). Pain levels, morphine equivalents, and 30-day surgical readmission rate did not differ between home-discharge passed-NERVS and non-NERVS groups.

**Conclusion:**

Our data demonstrates that nurse-driven mobilization in lieu of indiscriminate PT/OT evaluation after cranial tumor resection is associated with reduced hospitalization lengths-of-stay and total hospital charges among propensity-matched individuals, without an increase surgical readmission rate. Future mechanistic studies are necessary to determine if neurosurgical patients requiring less intensive post-operative rehabilitation assessment causally benefit from accelerated nurse-driven mobilization protocol.

**Supplementary Information:**

The online version contains supplementary material available at 10.1007/s00701-025-06641-1.

## Introduction

Surgical procedures often result in significant post-operative pain, physiological stress, extended hospital stays, reduced mobility, high risk of complications, and prolonged recovery times [5, 9, 11, 12]. To address these issues, methods for optimized peri-operative patient management have been explored across various surgical sub-specialties, frequently formalized into Enhanced Recovery After Surgery (ERAS) protocols. These protocols provide comprehensive recommendations across the continuum of care—pre-operative, operative, and post-operative periods—and have been shown to improve outcomes and optimize hospital resource allocation [2, 4, 5, 8, 10, 12].

In cranial neurosurgery, comprehensive ERAS protocols are less well-established due to the ongoing development of evidence supporting specific recommendations at each stage of the care continuum. Multiple institutional studies and comprehensive literature reviews have demonstrated that ERAS methods are associated with reduced post-operative pain, complications, and hospital length of stay (LOS) [3, 4, 8]. However, one area notably lacking in specific evidence-based recommendations is early post-operative mobilization.

Often, the responsibility for early post-operative mobilization falls on physical therapy and occupational therapy (PT/OT) services. PT/OT departments face an increasing demand for post-operative evaluations, with projected shortages expected to quadruple by 2030 [7, 13]. This trend aligns with our institutional experience, where PT/OT wait times frequently delay early post-craniotomy discharge. Recent spinal neurosurgical literature has shown that early mobilization protocols capable of bypassing PT/OT can safely reduce post-operative wait times [1].

Despite these promising results, there is no literature on the use of early nurse-driven mobilization protocols in cranial neurosurgical cases. In this study, we describe our experience with implementing our institution’s novel nurse-driven mobilization protocol (NERVS), which focuses on early post-operative movement, pain management, and patient education for cranial neurosurgical procedures. The aim of this study was to retrospectively compare resource utilization in patients following the NERVS protocol with non-NERVS controls.

## Methods

### Study design

We performed a retrospective observational cohort study of patients undergoing cranial tumor resection at our institution between 2017 and 2024, assessing patients who did or did not undergo the NERVS protocol post operatively. This study was approved by the local institutional review board (IRB #10–000655). Manuscript data was extracted in April 2024 and stripped of all protected and identifiable health information prior to analysis. Informed consent was exempted by the IRB as no personal identifying information is disclosed throughout this study.

### Patients

All patients with at least one qualifying Current Procedural Terminology (CPT) code (Table S1) for craniotomy for tumor resection between January 1 st 2017 and April 20th 2024 were initially included. Among these, patients were excluded if they were less than 18 years-old, were discharged on the same day as their cranial tumor resection, had a disqualifying diagnosis (primary spinal pathology, normal pressure hydrocephalus, or stereotactic radiosurgery treated-lesions) or required intravenous analgesic infusion for longer than one day post-operatively. Several relevant datapoints (Table S2) were extracted from the electronic medical record (EMR) for each patient in the final cohort.

### Assignment

All patients either entered or did not enter the NERVS protocol based on predefined criteria. Patients were assigned to NERVS if they underwent elective cranial tumor resection and were without pre- or postoperative neurological deficit. We performed statistical matching on several covariates to adjust for underlying population-level differences in the NERVS and non-NERVS subgroups before conducting comparative analyses. These covariates include: age, sex, procedure duration, ICU duration, readmission or not within 30 days, and case type (glioma, metastasis, meningioma, TNTS, other).

### Outcomes

Primary outcomes include total hospital LOS, total post-ICU LOS, total hospital billing, and total professional billing. Secondary outcomes included post-operative pain scores and analgesic dosing. Outcomes were compared between matched NERVS and non-NERVS subgroups. An additional analysis where patients staying in the ICU for longer than 24 h post-operatively were excluded.

### Statistical analysis

Data analysis was performed using MATLAB (Version R2023a). Two-sample t-tests were performed to compare quantitative values between two groups. All p-values < 0.05 were considered statistically significant. Specific criteria were used for patient assignment to NERVS: (1) no existing or post-operative neurological deficit (2) elective surgery (3) cranial patients. To eliminate additional confounders given the retrospective nature of our analysis, a propensity matching analysis was performed between those entering NERVS and those not entering NERVS. Matched covariates included age, sex, tumor type, procedure duration, ICU length-of-stay (LOS), and readmission to OR within 30 days or not. Multinomial logistic regression (MLR) was derived to predict likelihood of entering NERVS based on covariates; then, patients were matched using nearest neighbor matching with a standard caliper width of 0.2. Missing categorical datapoints were classified into an “Unknown/Other” category and are indicated in tables as such.

## Results

### NERVS protocol

**Fig. 1:**
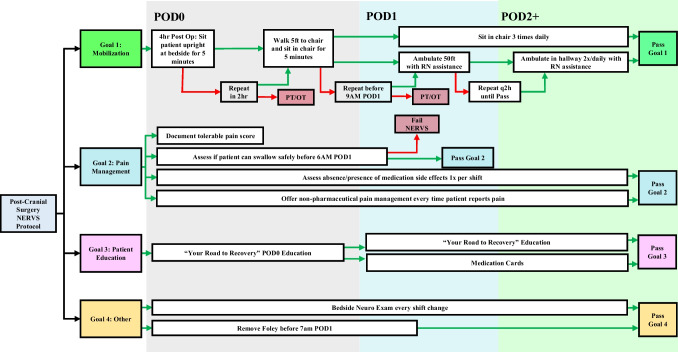
Flow Diagram of NERVS Protocol for four main goals. Patient is treated along 4 goals once entering the protocol. Green and red arrows indicate course of action if the preceding step is passed or failed, respectively. Once all 4 goals are achieved, a patient is considered to have passed the NERVS protocol. Red boxes indicate next step if the overall protocol is failed at a particular step, such as being shunted over to the traditional PT/OT evaluation prior to discharge. If a patient does not enter the NERVS protocol, they are assessed using the traditional evaluation from PT/OT prior to discharge

This work utilized the NERVS protocol, implemented at UCLA in 2013 for elective cranial neurosurgical cases. Developed in collaboration with neurosurgical faculty, residents, nurses, and anesthesiologists, this nurse-driven mobilization protocol focuses on three primary post-operative goals: i) mobilization, ii) pain management, and iii) patient education. Secondary goals include early Foley catheter removal and specific nursing guidelines. Regarding mobilization, on POD0, the protocol encourages sitting the patient upright for 5 min within 4 h of surgery, with repeated attempts every 2 h. This is followed by walking 5 feet to a chair, and on POD1 and beyond, sitting in a chair three times daily. Subsequent mobilization goals include walking 50 feet with RN assistance, then walking in the hallway with gradually less RN assistance. For pain management, POD0 tasks include a baseline pain assessment, a swallow study before POD1, and the implementation of non-pharmacological pain management. Patient education involves medication information cards, post-operative care instructions, and organizing clinical follow-up. Failure to achieve any NERVS goal initially results in re-attempt 6–8 h later. Additional failure on goals results in pathway failure and subsequent PT/OT evaluation for the traditional discharge pathway per hospital policy (Fig. 1).

### Patient selection workflow

All patients who had cranial tumor resections at UCLA between 2017 and 2024 were initially included (2719 patients). Exclusion criteria included same day discharge (*n* = 21), IV pain drip for more than one day (*n* = 115), disqualifying diagnoses (*n* = 121), age under 18 years (*n* = 105), multiple resections (*n* = 56), patient expiration (*n* = 4), and receipt of both NERVS and PT/OT orders at the same time (*n* = 703), resulting in a total of 1,594 included patients. These patients were then categorized into two groups based on whether they received the NERVS protocol: 1,059 patients received NERVS and 535 did not.

**Fig. 2:**
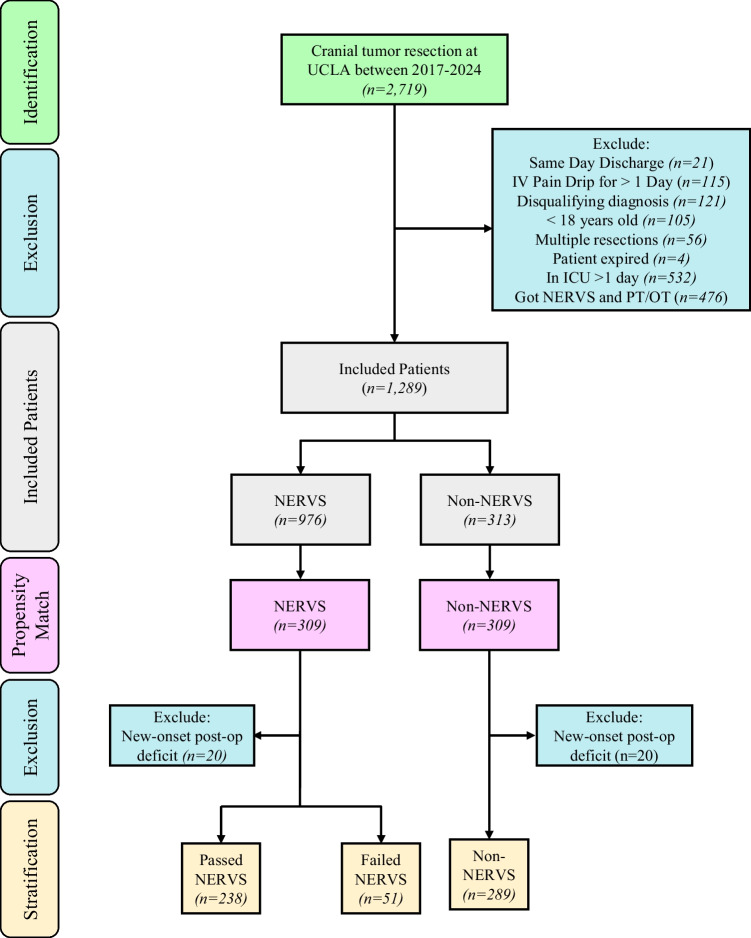
Inclusion flowchart for propensity-matched patients. ICD codes were utilized to identify an initial patient group. Subsequent exclusion criteria included: same-day discharge, IV pain drip for > 1 day post-operatively, harboring a disqualifying diagnosis, being < 18 years of age, having undergone multiple resections during the present hospitalization, having expired during the present hospitalization, having been in the ICU post-operatively for longer than one day, and having undergone NERVS and PT/OT during the present hospitalization. Patients were finally stratified into NERVS subgroups of passed, failed, or did not enter the NERVS protocol

To eliminate the confounding factors inherent in a retrospective analysis, a propensity match was performed on multiple core characteristics, yielding two groups of 309 patients each. Following this, 20 patients were excluded due to new-onset post-operative deficits. The final stratification included 238 patients who passed NERVS, 51 who failed NERVS, and 289 non-NERVS patients. This meticulous selection and stratification process ensures a balanced comparison for evaluating the impact of the NERVS protocol on surgical outcomes. Inclusion flowcharts for the initial cohort and propensity-matched patients are also shown in Figure S1 and Fig. 2, respectively**.**

### Full NERVS and non-NERVS group comparison

**Fig. 3:**
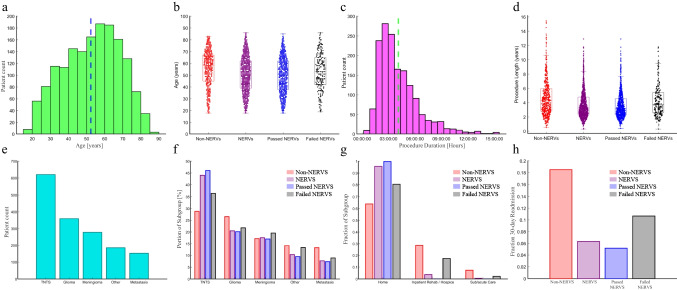
All patients (unmatched) cohort characteristics. Age distribution (**a**) cohort-wide and (**b**) by NERVS subgroup; Procedure duration distribution (**c**) cohort-wide and (**d**) by NERVS subgroup. Tumor type distribution (**e**) cohort-wide and (**f**) by NERVS subgroup; (**g**) discharge disposition by NRVS subgroup; (**h**) 30-day readmission rate distribution by NERVS subgroup

We summarize key characteristics of unmatched NERVS and non-NERVS groups in Table 1. The mean age of the overall cohort was 52.3 years, with non-NERVS being slightly older compared to NERVS (mean age 55.4 vs 50.7 years, *p* < 0.001). The NERVS group had a wider age range that skewing towards younger patients compared to the non-NERVS group. Gender and racial distributions showed no significant difference between groups. Procedure length showed no significant differences between our cohorts. ICU LOS and 30-day readmission rate both differed significantly between NERVS and non-NERVS groups. Tumor type distribution indicates a higher proportion of patients with pituitary tumors (TNTS) in the NERVS group compared to the non-NERVS group (44.0% vs 28.8%, *p* < 0.001). Other tumor types such as glioma, meningioma, metastasis, etc. do not show statistically significant differences between the groups. Graphical summaries of full-group comparisons are shown in Fig. 3. A total of 1355 (85%) of patients went home, 193 (12.1%) went to inpatient rehab/hospice, and 46 (2.9%) went to acute or subacute care. Given the noted baseline differences between the NERVS and non-NERVS groups, it was necessary to perform a propensity-based match on demographic and clinical characteristics before performing LOS and cost comparisons.

**Table 1:** Demographics and admission profiles of all included patients

	All	Subgroups		
		No NERVS	NERVS	*p*-value
Count	1594	535 (33.6%)	1059 (66.4%)	-
Age (Years)				
Mean (SD)	52.3 (± 15.5)	55.4 (± 15.6)	50.7 (± 15.3)	<.001*
Median (Q1-Q3)	53 (40—65)	58 (45—67)	51 (39—62)	-
Age category				
< 18	0	0	0	-
18 to < 50 years	658 (41.3%)	175 (32.7%)	483 (45.6%)	<.001*
50 to < 65 years	537 (33.7%)	183 (34.2%)	354 (33.4%)	0.76
≥ 65 years	399 (25.0%)	177 (33.1%)	222 (21.0%)	<.001*
Sex				
F	813 (51.0%)	258 (48.2%)	555 (52.4%)	0.11
M	781 (49.0%)	277 (51.8%)	504 (47.6%)	
Race				
Non-Hispanic White	809 (50.8%)	277 (51.8%)	532 (50.2%)	0.56
Hispanic	353 (22.1%)	113 (21.1%)	240 (22.7%)	0.48
Asian Pacific Islander	207 (13.0%)	69 (12.9%)	138 (13.0%)	0.94
Non-Hispanic Black	93 5.8%)	36 (6.7%)	57 (5.4%)	0.28
American Indian/Alaskan Native	4 (0.3%)	2 (0.4%)	2 (0.2%)	0.49
Unknown/Other	128 (8.0%)	38 (7.1%)	90 (8.5%)	0.33
Procedure length (Hours)				
	4.1	4.0	4.2	0.12
ICU stay duration (Hours)				
	26.0	56.1	10.8	<.001*
30-day readmission rate				
	0.10	0.19	0.06	<.001*
Tumor type				
Pituitary (TNTS)	620 (38.9%)	154 (28.8%)	466 (44.0%)	<.001*
Glioma	359 (22.5%)	142 (26.5%)	217 (20.5%)	0.006
Meningioma	278 (17.4%)	92 (17.2%)	186 (17.6%)	0.86
Metastasis	152 (9.5%)	71 (13.3%)	81 (7.6%)	<.001*
Other	185 (11.6%)	76 (14.2%)	109 (10.3%)	0.02

^*^missing data indicated as “Unknown/Other” or “Other” when present

### Propensity-based matched analysis

**Fig. 4:**
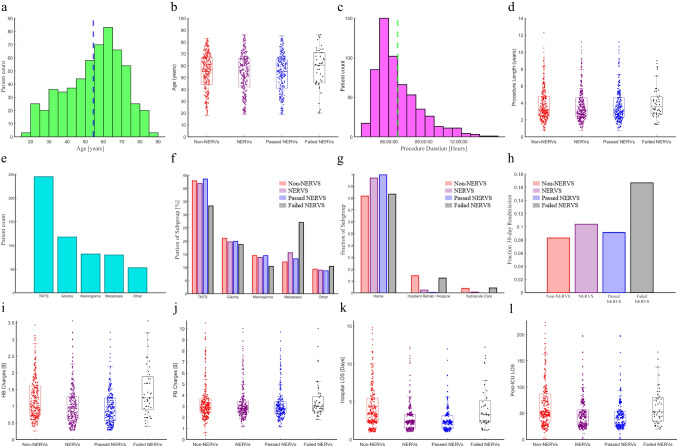
Matched cohort characteristics. Age distribution (**a**) cohort-wide and (**b**) by NERVS subgroup. Procedure duration distribution (**c**) cohort-wide and (**d**) by NERVS subgroup. Tumor type distribution (**e**) cohort-wide and (**f**) by NERVS subgroup. (**g**) Discharge disposition by NERVS subgroup. (**h**) 30-day readmission rate distribution by NERVS subgroup. (**i**) Hospital billing (HB) charges and (**j**) professional billing (PB) charges by NERVS subgroup. (**k**) Hospital length-of-stay and (**l**) post-ICU length-of-stay by NERVS subgroup

We summarize key characteristics of matched NERVS and non-NERVS groups in Table 2. The propensity-matching algorithm yielded two groups of 309 patients each. As discussed, 20 patients were excluded due to new post-operative deficits. Since new post-op deficits invalidate the criteria of patients being matched on every possible characteristic, these pairs were eliminated in order to allow for the most balanced evaluation of the effect of the NERVS protocol on hospital length of stay, discharge, and cost. Our final stratification included 238 patients who passed NERVS, 51 who failed NERVS, and 289 non-NERVS patients. All figures for the unmatched cohort were re-created for the short-ICU matched cohort (Fig. 4).

**Table 2:** Demographics and admission profiles of matched cohort

	All	Subgroups		
		No NERVS	NERVS	p-value
Count	578	289 (50%)	289 (50%)	-
Age (Years)				
Mean (SD)	54.4 (± 15.8)	55.1 (± 15.4)	53.7 (± 16.3)	0.82
Median (Q1-Q3)	57 (43—66)	57 (44—67)	56 (42—66)	-
Age category				
< 18	0	0	0	-
18 to < 50 years	199 (34.4%)	94 (32.5%)	105 (36.3%)	0.34
50 to < 65 years	211 (36.5%)	109 (37.7%)	102 (35.3%)	0.55
≥ 65 years	168 (29.1%)	86 (29.8%)	82 (28.4%)	0.71
Sex				
F	281 (48.6%)	142 (49.1%)	139 (48.1%)	0.80
M	297 (51.4%)	147 (50.9%)	150 (41.9%)	
Race				
Non-Hispanic White	303 (52.4%)	154 (53.3%)	149 (51.6%)	0.68
Hispanic	113 (19.6%)	55 (19.0%)	58 (20.1%)	0.75
Asian Pacific Islander	80 (13.8%)	40 (13.8%)	40 (13.8%)	1
Non-Hispanic Black	27 (4.7%)	16 (5.5%)	11 (3.8%)	0.39
American Indian/Alaskan Native	3 (0.5%)	2 (0.7%)	1 (0.3%)	0.56
Unknown/Other	52 (9.0%)	22 (7.6%)	30 (10.4%)	0.24
Procedure length (Hours, 0% missing)				
	3.8	3.6	3.9	0.08
ICU stay duration (Hours)				
	5.4	5.3	5.4	0.93
30-day readmission rate				
	0.09	0.08	0.10	0.39
Tumor type				
Pituitary (TNTS)	245 (42.4%)	124 (42.9%)	121 (41.9%)	0.80
Glioma	118 (20.4%)	61 (21.1%)	57 (19.7%)	0.68
Meningioma	82 (14.2%)	42 (14.5%)	40 (13.8%)	0.81
Metastasis	80 (13.8%)	35 (12.1%)	45 (15.6%)	0.23
Other	53 (9.2%)	27 (9.3%)	26 (9.0%)	0.89

^*^missing data indicated as “Unknown/Other” or “Other” when present

Demographically (age, gender, race), we note no significant differences between matched non-NERVS and NERVS groups. The mean age of the overall cohort is 54.4 years (55.1 years non-NERVS, 53.7 NERVS, *p* = 0.82). Gender distribution also reflects no significant difference. Racial composition is evenly distributed, with the majority of patients being Non-Hispanic White (52.4%). Clinically, procedure length and tumor type distribution were balanced between the two groups. The average procedure length is 3.8 h for the overall cohort, with non-NERVS and NERVS procedures averaging 3.6 and 3.9 h respectively (*p* = 0.08). Tumor type distributions were statistically similar across all types, with pituitary tumors (TNTS) being the most common in both groups and accounting for 42.4% of the overall cohort. Importantly, there was no longer a difference in post-op ICU LOS (5.3 h non-NERVS, 5.4 h NERVS, *p* = 0.93).

Among short-ICU propensity-matched cohort, differences in LOS and costs remained significant. Hospital LOS was shorter among NERVS patients vs non-NERVS patients (2.9 vs 4.6 days, *p* < 0.001). This corresponded with a lower total HB among NERVS patients ($104,050 NERVS vs $130,090 non-NERVS, *p* < 0.001) and similar total PB ($33,530 NERVS, $35,090 non-NERVS, *p* = 0.33). Furthermore, post-operative LOS (2.3 days NERVS, 3.1 days non-NERVS, *p* < 0.001) and post-ICU LOS (2.1 days NERVS, 2.9 days non-NERVS, *p* < 0.001) were also significantly shorter among NERVS patients.

In summary, the propensity-matched cohort analysis demonstrates no significant demographic or initial clinical differences between non-NERVS and NERVS patients, validating the matching process. This allows for a focused comparison of clinical outcomes and resource utilization between the two groups, with our results suggesting key differences in discharge disposition, length of stay, and associated charges. Graphical summaries of propensity-matched comparisons are shown in Fig. 4. Overall, there were an estimated 300 PT and 292 OT consults saved across our cohort of 2,297 patients.

## Discussion

In this observational cohort study, we found that enrollment in the described nurse-driven early post-cranial tumor resection mobilization protocol in lieu of PT/OT was associated with significantly reduced hospital LOS by 1.7 days (*p* < 0.001), post-operative LOS by 0.8 days (*p* < 0.001), post-ICU LOS by 0.8 days (*p* < 0.001), and total hospital billing by over $26,000 (*p* < 0.001) per patient. Enrollment in NERVS or not did not significantly alter post-operative pain levels or analgesic use (Figure S2). These findings, though associative, suggest that cranial neurosurgical patients requiring less intensive post-operative rehabilitation assessment may benefit from accelerated nurse-driven mobilization protocols without a formal PT/OT consultation. Mechanistic studies are necessary to explore the causal nature of this association.

Our results agree with findings from other studies on ERAS pathways, both the limited studies in cranial surgical populations and broader studies performed in other groups. ERAS methods are associated with reduced post-operative pain, complications, and hospital length of stay [2, 4, 6, 8]. There is specifically data in spinal neurosurgery populations that early physical therapy can improve patient recovery [1]. However, most existing ERAS protocols for cranial procedures have not extensively focused on post-operative mobilization​​. This highlights a significant gap in the literature that our study aims to fill. Here, we show that in a cranial neurosurgical population, early post-operative mobilization is an important component of the ERAS protocol and is associated with reduced length of stay without change in complication rates. One possible contributor to the perceived difference in LOS between NERVS and non-NERVS groups is the time spent determining disposition in non-NERVS patients, which is presumably shorter in NERVS patients.

Our inclusion and exclusion criteria were carefully designed to capture elective craniotomy patients without post-operative deficits who were appropriate for the NERVS protocol from a variable population of all craniotomy patients in our institution over the past five years. Notably, in our study, there were specific criteria used to identify whether a patient was appropriate for the NERVS protocol. Despite that, on our analysis, we did note that there was a large percentage of our population that was receiving orders for both NERVS and PT/OT simultaneously. Upon further review of these patients, these orders were driven by specific clinician judgement or family request. These patients were not included in our analysis as they did not follow the traditional NERVS pathway. However, this observation does highlight the need for consistency in practice decisions.

In addition, we filtered patients based on length of stay to capture patients who followed the general pathway of elective craniotomy without post-operative deficit. Thus, patients who were discharged on the same day as their procedure were not included as they were not admitted and thus did not qualify for NERVS protocol. In the same vein, patients who had prolonged stays in the ICU with ongoing IV drip pain medications were also not included as they do not represent a population appropriate for the NERVS protocol.

Notably, in our analysis, approximately 60% of NERVS patients passed the protocol immediately while 40% took as long as to pass as a PT/OT consult takes to be conducted. This highlights the efficiency of the NERVS protocol in expediting patient recovery and discharge. It additionally highlights the increased efficiency that would be possible for our PT/OT colleagues by eliminating unnecessary consults through this protocol. Identification of patients who truly need PT/OT with this protocol could allow for patients who require services to be seen in an expedited fashion. Because the protocol of not ordering PT/OT concurrently with NERVS was not always strictly enforced in this context, our estimates for saved number of PT/OT consults (300 and 292, respectively) likely underestimate true savings. Regardless, PT/OT costs are bundled, and their reduction drives an overall decrease in price as empirically observed in this study.

## Limitations

This study is subject to limitations of single-center and retrospective analyses. At other institutions, differing frequencies of procedure types, available staffing, and patient populations, to name a few, may limit the generalizability of these findings. With non-random assignment to NERVS or not, population level differences may exist between the NERVS and non-NERVS groups. We performed a propensity matched analysis on perceived confounds to mitigate these effects. Nonetheless, prospective studies are necessary to validate these findings. Furthermore, NERVS enrollment guidelines were not correctly implemented in all patients, resulting in a subset of patients completing NERVS and/or PT/OT in the incorrect order. As discussed, these patients were excluded from this study and serve as valuable case studies to be examined for iterative improvement of protocol execution at our institution. Finally, because propensity matching reduced the study population considerably, these findings may not reflect our entire cranial surgery population.

## Conclusion

This study revealed that our cranial ERAS pathway involving early nurse-driven mobilization after cranial tumor resection was associated with significantly reduced post-operative length-of-stay and total hospital billing in the matched cohort. These findings agree with previously reported evidence in the spinal neurosurgery literature and suggest that PT/OT evaluation prior to discharge may not be necessary in all patients after cranial neurosurgery. Although prospective and multi-center mechanistic validations of our conclusions are necessary, this study provides specific recommendations on, and details cost savings associated with an early mobilization protocol after cranial tumor resection.

## Supplementary Information

Below is the link to the electronic supplementary material.

Supplementary file1 (PDF 17.1 KB)

Supplementary file2 (PDF 218 KB)

Supplementary file3 (DOCX 14 KB)

Supplementary file4 (DOCX 14 KB)

## Data Availability

No datasets were generated or analysed during the current study.
