# Supplementary figures and images for: Cost savings associated with a nurse driven mobilization protocol for recovery after cranial tumor resection
Source: Acta Neurochir (Wien). 2025 Sep 2;167(1):237. doi: 10.1007/s00701-025-06641-1 (PMC12405358; doi:10.1007/s00701-025-06641-1)

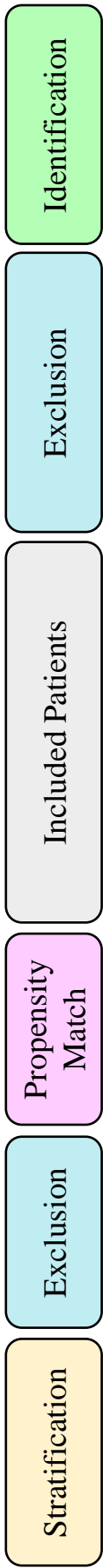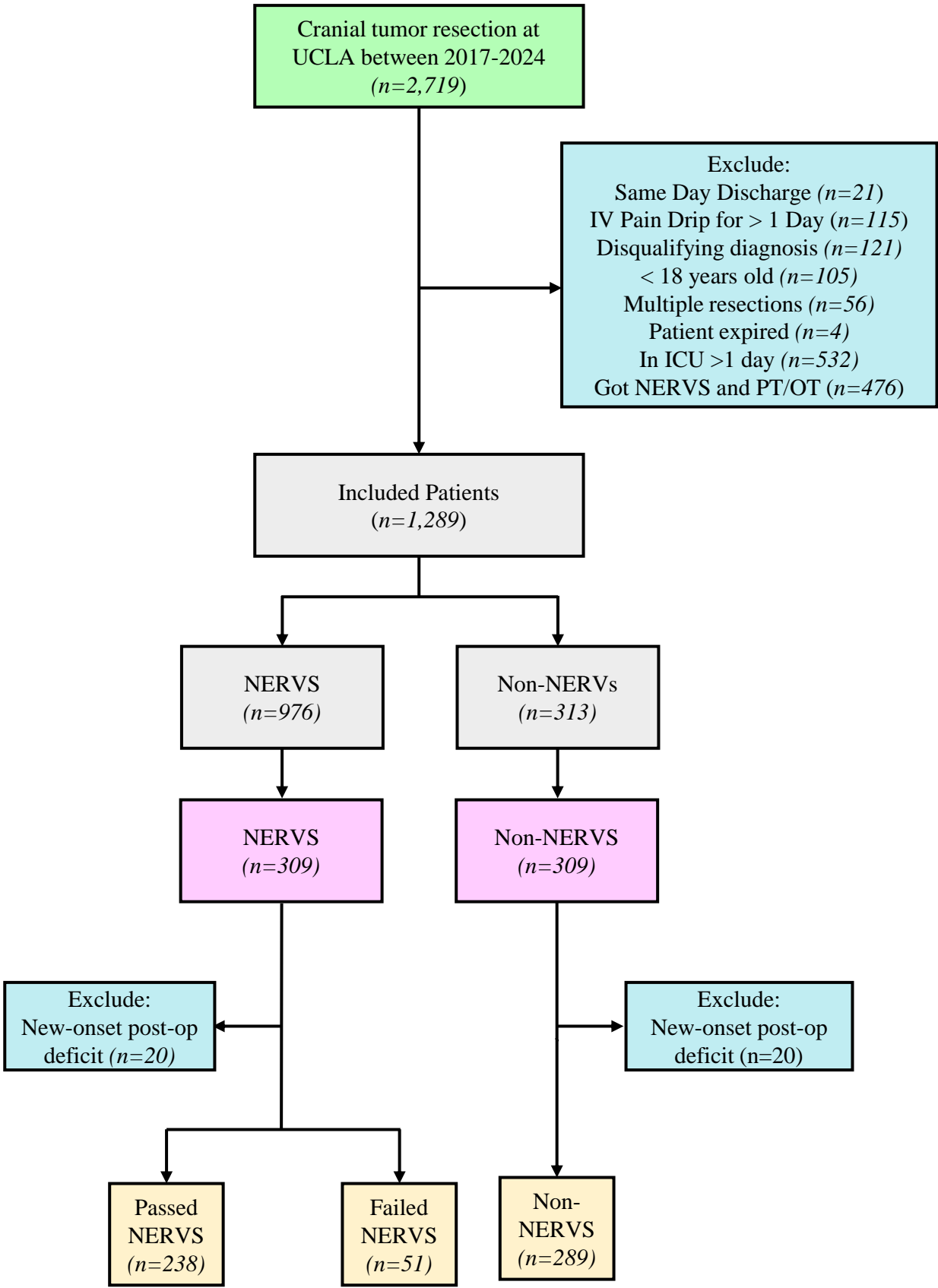

Supplement: Supplementary file 1 — Supplementary file1 (PDF 17.1 KB) [file 701_2025_6641_MOESM1_ESM.pdf]

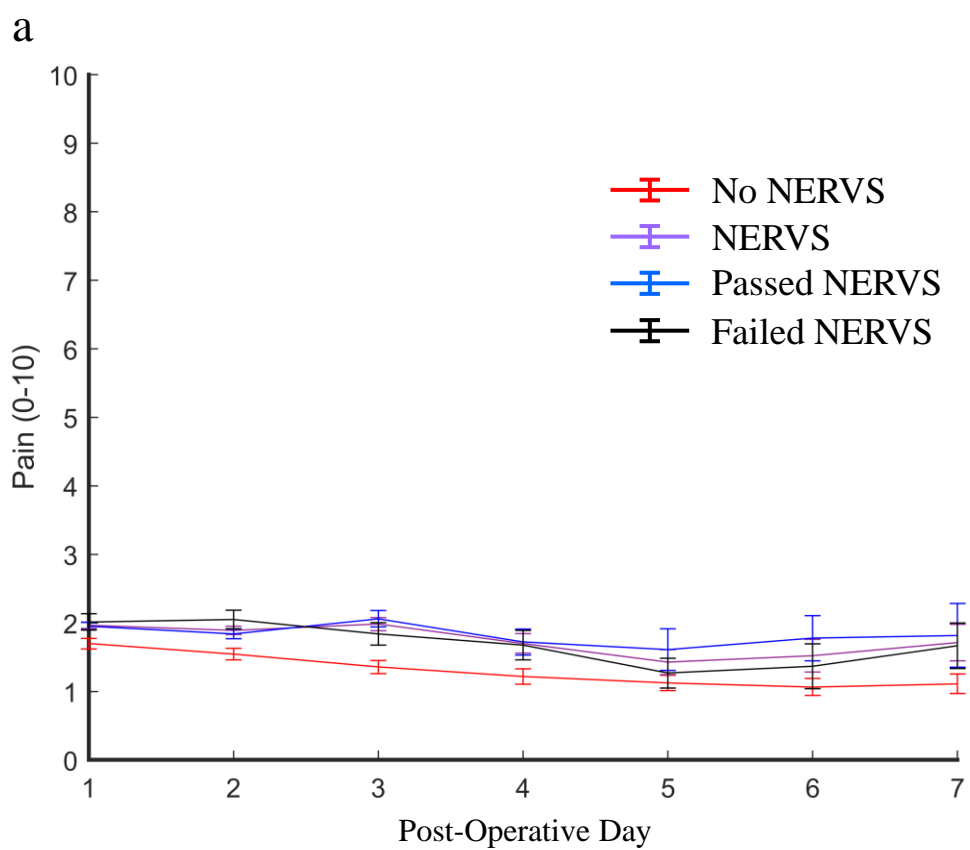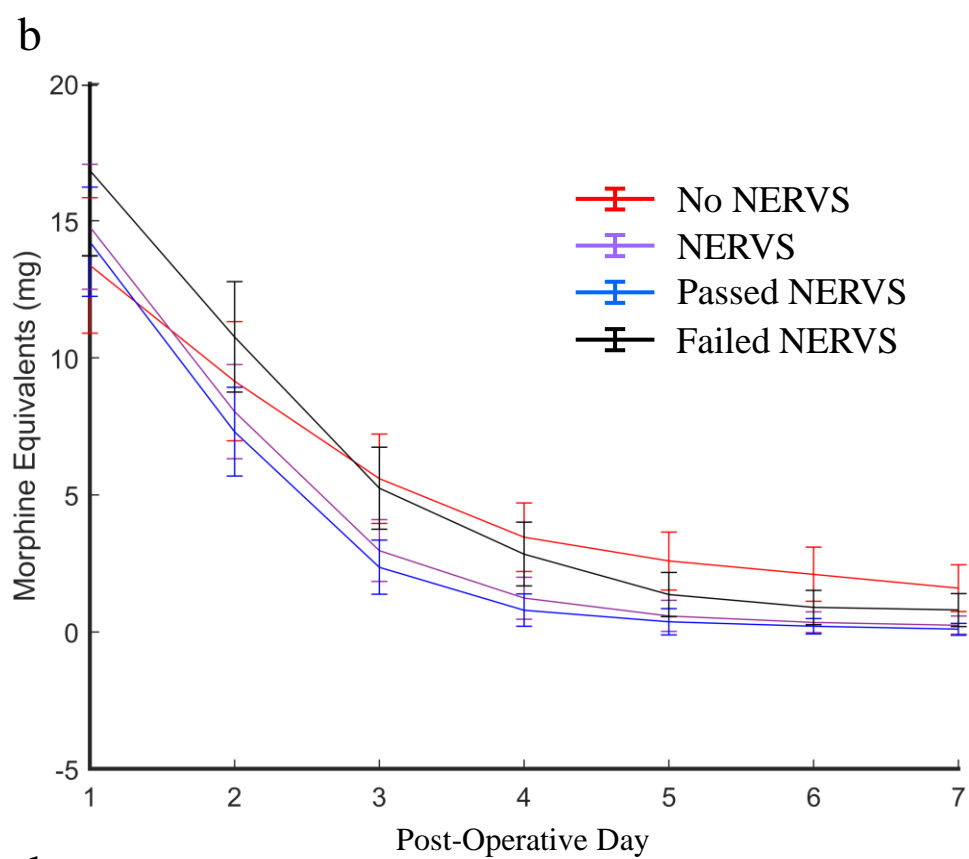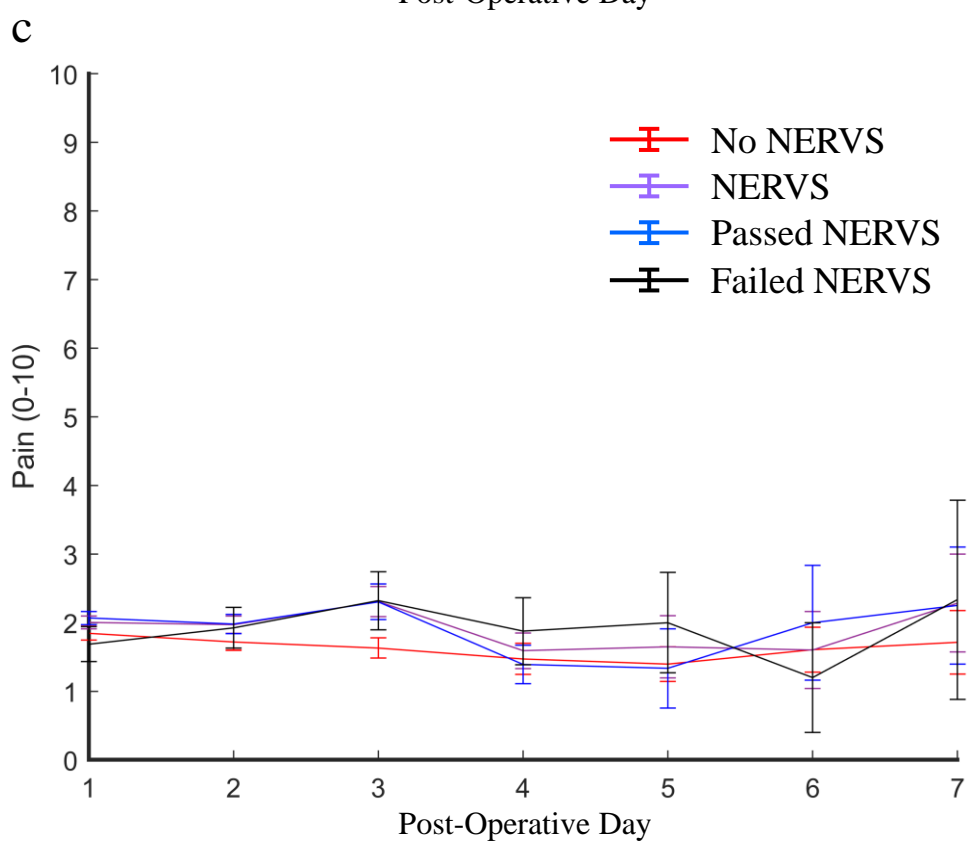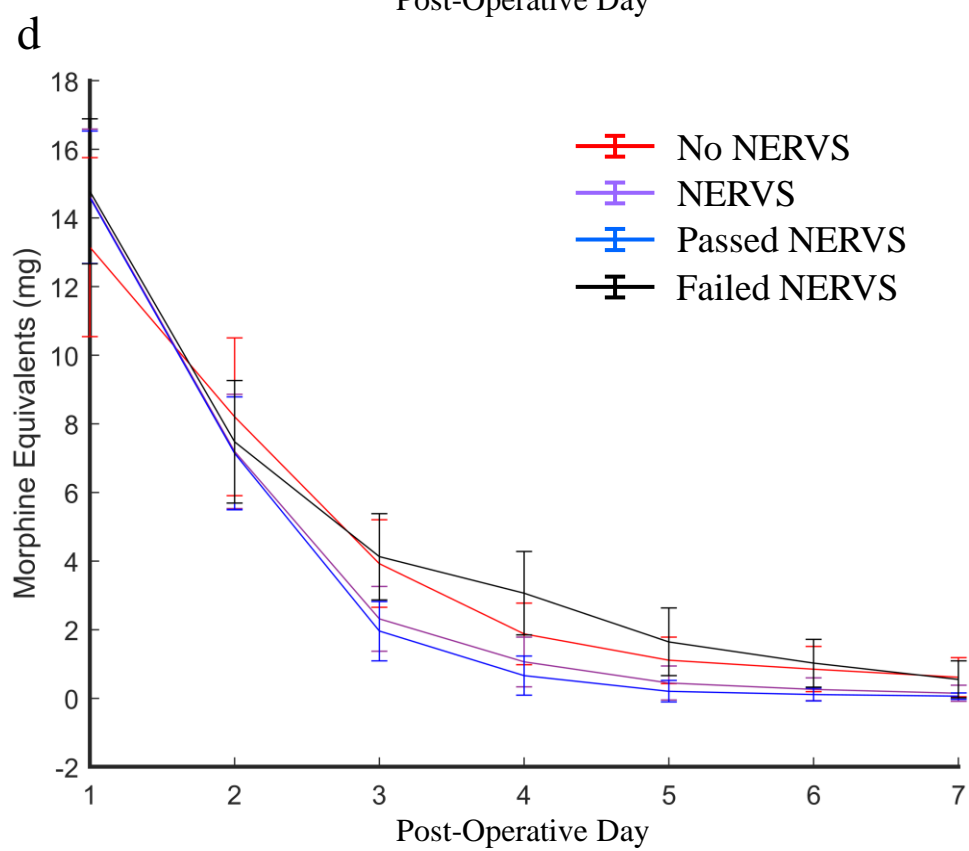

Supplement: Supplementary file 2 — Supplementary file2 (PDF 218 KB) [file 701_2025_6641_MOESM2_ESM.pdf]
