# Supplementary material for: Cost savings associated with a nurse driven mobilization protocol for recovery after cranial tumor resection
Source: Acta Neurochir (Wien). 2025 Sep 2;167(1):237. doi: 10.1007/s00701-025-06641-1 (PMC12405358; doi:10.1007/s00701-025-06641-1)
Supplement: Supplementary file 3 — Supplementary file3 (DOCX 14 KB) [file 701_2025_6641_MOESM3_ESM.docx]

| CPT Code | Description |
| --- | --- |
| 61510 | EXCIS SUPRATENT BRAIN TUMOR |
| 61512 | EXCIS SUPRATENT MENINGIOMA |
| 62165 | NEUROENDOSCOP,EXC,PIT TUM,TRANSNAS/SPHEN |
| 61518 | EXCIS INFRATENT BRAIN TUMOR |
| 61519 | EXCIS INFRATENT MENINGIOMA |
| 61520 | EXCIS INFRATENT CP ANGLE TUMOR |
| 61521 | EXCIS INFRATENT MIDLINE TUMOR |

**Table S1**. List of initial inclusion CPT codes.
