# Supplementary material for: Cost savings associated with a nurse driven mobilization protocol for recovery after cranial tumor resection
Source: Acta Neurochir (Wien). 2025 Sep 2;167(1):237. doi: 10.1007/s00701-025-06641-1 (PMC12405358; doi:10.1007/s00701-025-06641-1)
Supplement: Supplementary file 4 — Supplementary file4 (DOCX 14 KB) [file 701_2025_6641_MOESM4_ESM.docx]

| Age |
| --- |
| Sex |
| Ethnicity |
| Race |
| Hospital admission time |
| Hospital discharge time |
| Perioperative diagnosis |
| Surgery start time |
| Surgery end time |
| Discharge disposition  Home  Inpatient Rehab / Hospice  Acute Care  Subacute Care  Expired |
| Hours in ICU |
| Readmission within 30 days (Yes/No) |
| PT/OT Ordered (Yes/No) |
| Entered NERVS |
| Passed NERVS (Yes/No) |
| Total Professional Billing ($) |
| Total Hospital Billing ($) |
| Procedure category  Glioma resection  Metastasis resection  Meningioma resection  Endoscopic pituitary tumor resection  Other |
| Post-operative Pain Scores |
| Post-operative Analgesic Dosing |

**Table S2**. EMR fields extracted for each patient.
